# Anticipation of pain during operant learning increases cognitive performance and feedback-related cortical potentials

**DOI:** 10.1007/s00221-025-07167-9

**Published:** 2025-10-04

**Authors:** Carolina Ceruti, Laura Petrini, Giulia Erica Aliotta, Dennis Boye Larsen, Elia Valentini, Kristian Hennings, Carina Graversen, Carsten Dahl Mørch

**Affiliations:** 1 Department of Health Science and Technology, Faculty of Medicine, Center for Neuroplasticity and Pain (CNAP), Aalborg University, Aalborg, Denmark; 2 Department of Psychology, Centre for Brain Science, University of Essex, Colchester, UK

**Keywords:** Pain, Operant conditioning, Reward, Punishment, Electroencephalography, Event related potentials

## Abstract

Operant conditioning (OC) evokes behavioral changes and may be useful in pain management. However, it is unknown how alteration of a tonic painful stimulus may affect cognitive performance in an OC learning task and the associated neural activity. To address this, specific event-related potentials (ERPs) and cognitive performance were assessed after an OC task, using altered pain intensity as the operant stimulus. Two OC paradigms were designed using painful tonic pressure pain as the conditioning stimulus. 29 healthy participants received individually set tonic pressure pain corresponding to visual analogue scale 5 (VAS5; pain threshold). Pressure was maintained and a cognitive task performance yielded reward or punishment. Consequences of correct and incorrect answers in the negative reinforcement (NR) condition were pain relief (VAS3) or no pressure change (VAS5), respectively, and no pressure change (VAS5) or increased pressure (VAS7) in the positive punishment (PP) condition, respectively. The initial condition (NR or PP) was randomized, and 120 trials were conducted in three same-day sessions. 64-channel electroencephalography was recorded, and auditory-feedback ERPs (P1N1, P2N2, P3N3) were extracted. Higher ERP peak-to-peak amplitudes were found when participants received feedback that their answer was incorrect. A small OC learning behavior effect was found across trials with no difference between NR and PP. Independent of OC paradigm, learning behavior was induced, and ERP complex amplitudes increased when incorrect answers were given. These novel findings show that higher pain expectancy due to an incorrect answer, facilitated feedback-related ERPs when using pain as a conditioning stimulus.

## Introduction

Learning from appetitive or aversive events is an important adaptive mechanism and according to the operant conditioning (OC) theory, behavior is shaped by its consequences (Staddon and Cerutti 2003). Traditionally, classical behavioral studies examined how reinforcement and punishment influence pain behaviors through OC (Staats et al. 1996). These studies suggested that reinforcement conditions (e.g. removing adverse stimulus; negative reinforcement (NR) strengthen behavior, while punishment conditions (e.g. adding adverse stimulus; positive punishment (PP) weaken behavior (Skinner 2014). Moreover, the Fordyce behavioral pain model (Fordyce 1984; Gatzounis et al. 2012) framed associative learning benefits in chronic pain conditions, highlighting the importance of multimodal approaches to pain management. For example, earlier studies demonstrated that OC can (i) modulate somatosensory processing in chronic back pain patients (Flor et al. 2002), (ii) reduce disability and pain in chronic low back pain patients (Bunzli et al. 2011), (iii) decrease experimental mechanical pinprick pain (Lee et al. 2021), and (iv) influence how pain intensity is reported (Linton and Gotestam 1985). Moreover, pain perception influences OC, where e.g. painful electrical stimulations decreased reward responses in purchase tasks (Ma et al. 2022), and clinically, patients with depression exhibited lower feedback event-related potential (ERP) amplitudes, when compared to healthy controls or no pain conditions (Whitton et al. 2016). Therefore, pain may affect reward processing, by blunting the reward responsiveness that can be captured using brain activity measures such as ERPs and electroencephalography (EEG). This method allows for identifying event-specific peaks that could reflect different stages of information processing (Luck 2005). For example, the N2 component is sensitive to reinforcement learning (Glazer et al. 2018) and is largely driven by reward prediction error (Heydari and Holroyd 2016), that reflects differences in gains and losses, though salience prediction error has also been implicated (Glazer and Nusslock 2023; Talmi et al. 2013). Interestingly, generation of reward positivity becomes absent or reduced when pain feedback is received (Heydari and Holroyd 2022), indicating that experimental painful settings mimic the clinical manifestation of blunted reward responses. Additionally, an increase in P2 and P3 amplitudes is observed when an absolute reward is given (Wischnewski and Schutter 2018), and based on motivational significance of a feedback stimulus (Wu and Zhou 2009), and stimulus-evoked P2 and P3 are increased with painful versus nonpainful stimuli (Willems et al. 2021). In the context of monetary reward, the largest increases in N2 and P3 amplitudes were found in monetary loss (punishment condition), rather than monetary gain (De Pascalis et al. 2010; Pfabigan et al. 2014). In the context of pain, it is less clear which role OC has on EEG processing. In this respect, the situation (e.g. being in pain) influences human behavior where feedback signals (e.g. auditory) of an outcome help create internal prediction models for reward and punishment which can be captured using electrophysiological methods (Tversky and Kahneman 1992; Wischnewski and Schutter 2018). It is, however, unknown how being in pain while receiving operant feedback on task performance influences early ERPs given their ability to capture the integration of both attentional engagement and cognitive control processes (Folstein and Van Petten 2008; Luck 2005; Polich 2007). Therefore, this study aimed to investigate the impact of performance feedback on operantly controlled auditory ERPs, during constant pressure pain, and hypothesized that auditory feedback ERPs would decrease when receiving negative feedback on the hearing task performance.

## Materials and methods

### Participants

29 healthy individuals (mean age 26.25 ± 5.29; 20 women) were included in the study. Exclusion criteria were a history of chronic or ongoing acute pain, neurological (e.g. diabetes, epilepsy, or Parkinson’s disease), musculoskeletal or mental (e.g. depression or stress) diseases, were addicted to drugs such as cannabis or opioids, or pregnancy. As the study used auditory feedback, participants were also excluded if they did not have normal hearing. Participants informed if they had hearing issues, but as the hearing task was calibrated individually, a specific hearing threshold was not necessary for the conduct of the experiment. All participants signed an informed consent form and received monetary compensation at the end of the experimental session (150 DKK for each hour spent in the laboratory). The study was approved by the local ethics committee N-20220014 and was in accordance with the standards of the declaration of Helsinki.

### Experimental design and procedure

**Fig. 1:**
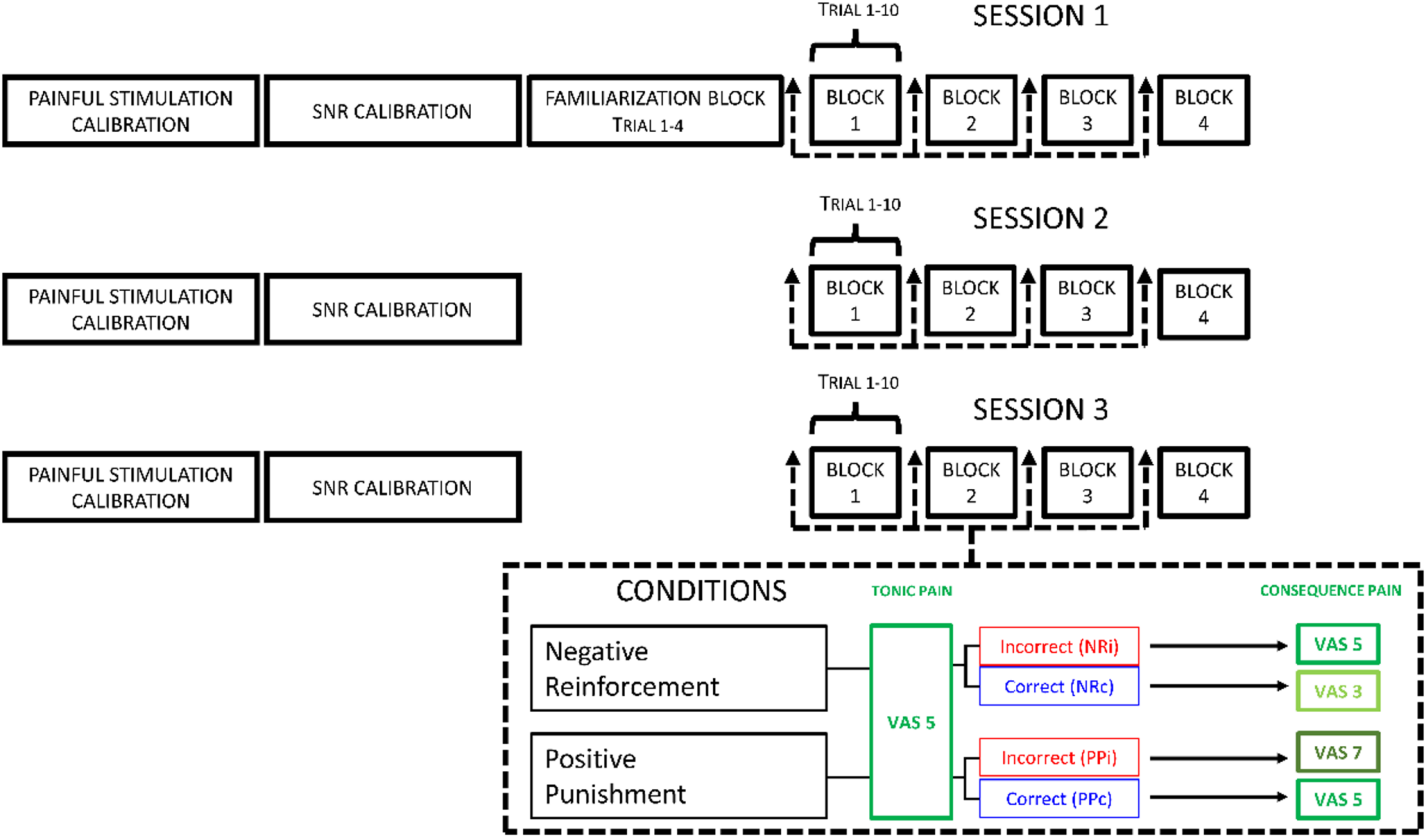
Experimental set-up. Every participant went through 120 trials divided into 3 sessions made of 4 blocks, each with 10 trials. At the beginning of every block the participant was presented to the condition (Negative reinforcement or Positive Punishment). Prior to each session, participants went through calibration of the painful stimulation and calibration of the SNR (sound-to-noise ratio). Before starting the experiment, a familiarization block of four trials was presented to participants. The pain level during the cognitive task (noise + sentences) was kept constant, while the consequence of task performance was split into two conditions. In the negative reinforcement condition, the subject experienced pain relief as a reward and unaltered pain stimulus as a punishment. In the punishment condition, the subject experienced increased pain as a punishment and unaltered pain stimulus as a reward

**Fig. 2:**
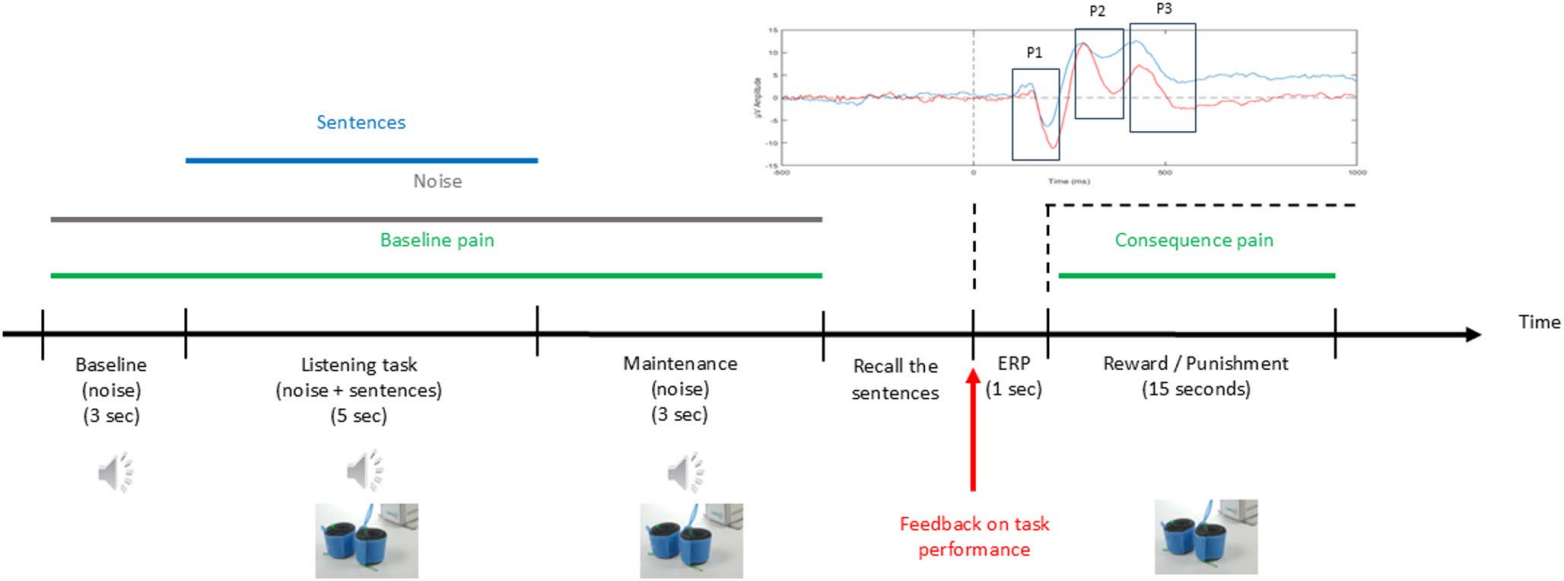
Experimental set-up of each trial. Each trial consisted of a baseline with the subject exposed to noise, followed by a listening task with HINT sentences. The HINT sentence consisted of five words, which the subject needed to memorize during the maintenance phase. After the maintenance phase, the subject was asked to recall the words, and based on the performance, feedback was provided with two different ‘beep’ sounds, indicating if the participant answered right or wrong, and hence if a reward or punishment was given. HINT: Hearing-In-Noise-Test

The experiment consisted of 120 trials in total divided into 3 sessions, all performed on the same day, separated by a 15 min break. Each experiment lasted a maximum of four hours. Each session consisted of 4 blocks with 10 trials in each block (Fig. 1). Four training trials were conducted at the beginning of the experiment to familiarize the participants with the methods and ratings, with both conditions presented twice. EEG recordings were acquired during the whole experiment. Each participant was randomized to start in two different operant conditioning paradigms, (1) negative reinforcement (NR) or (2) positive punishment (PP), which yielded operant control of the performance of the cognitive task. The participant was presented with the condition on-screen, at the start of the block. Within the sessions, each block was randomized to be either NR or PP, with two presentations of each. After the auditory presentation of sentences, three seconds of maintenance followed after which the participant recalled the sentences. Their performance determined if the participants received reward or punishment after task completion. During each trial, a painful stimulation, fixed to elicit pain of 5 on a visual analogue scale (VAS), was delivered while participants were performing an auditory task (10 s pressure stimulation). For the NR condition, a correct answer (i.e. correct performance leading to reward) yielded pain relief (VAS3) while an incorrect answer (i.e. incorrect performance leading to punishment) was considered unaltered pain (VAS5). In the PP condition, reward was considered unaltered pain (VAS5), while punishment was an increase in pressure pain (VAS7). Feedback was given to the participants in the form of two different auditory stimuli, each lasting one second, where one indicated a correct answer and one indicated an incorrect answer, where outcomes were auditory ERPs as measured by EEG. The auditory stimulus for a correct answer was a high-pitched tone, while the auditory stimulus for an incorrect answer was a low-pitched tone. Behavioral data on the number of correct and incorrect answers (and their related feedback) were extracted to assess the operant conditioning effect on performance of the auditory task. A schematic illustration of the experimental trial is shown in Fig. 2.

### Auditory task: hearing-in-noise-test

The auditory task consisted of a hearing-in-noise-test (HINT) where subjects were asked to repeat a sentence after hearing it. Sentences were set at a speech repetition threshold (SRT) of 50% with the purpose of obtaining a performance score of 50% (balanced numbers of incorrect and correct trials). To obtain the 50% balance between correct/incorrect answers, a signal to noise ratio (SNR) calibration was done prior to every session for every subject. The speech material consisted of 250 Danish HINT sentences (Nielsen and Dau 2011) and were presented diotically by earphones. The sentences were fully randomized for the listening effort. The experimenter scored the number of correct words in the sentence that the participant repeated after the listening effort and then gave feedback. To obtain a reward the participant had to repeat the complete sentence (made of 5 words) correctly, otherwise he/she would receive a punishment. Although the use of auditory feedback in our methods may resemble classical conditioning, the paradigm is based on modifying behavior through consequences, which defines it as operant conditioning (Staddon and Cerutti 2003). The experimenter was always present in the room during the auditory task performance.

#### Signal-to-noise ratio (SNR) calibration

This calibration consisted of estimating the psychometric function for the SNR using the Psi-Method (Kontsevich and Tyler 1999) as implemented in the Palamedes Toolbox (Prins and Kingdom 2018). The Psi-Method is an adaptive Bayesian method to estimate psychometric functions in relation to psychophysics. This method aims to optimize the number of trials for estimation and entropy minimization based on posterior distribution updating that yields the most informative stimulus intensity level for each threshold estimation. Based on the estimated psychometric function, the SNR level was set to the level that was estimated to result in a 50% probability of a correct response. The noise was a continuous white noise, used as a disturbance for the hearing of the sentences.

### EEG recording

64 electrodes (gTEC.hiAMP) were placed on the scalp of the participant according to the 10–20 international system. The impedance of the electrodes was kept under 10 kΩ, the signal was amplified and sampled with a sampling frequency of 1200 Hz (g.HIamp biosignal amplifier g.Tec medical engineering GmbH, Schiedlberg, Austria). The ear lobes were used as linked reference, and the ground was placed on AFz. Several triggers enabled correct alignment between pain and auditory stimulations and the EEG recordings. Each auditory feedback stimulus (correct or incorrect performance) elicited ERPs that were extracted − 500 ms before and 1000 ms after the tone was presented.

### Painful stimulation

The subjects were exposed to tonic painful stimulation applied by a tourniquet cuff (a silicon high-pressure cuff, Nocitech Aps) to deliver sustained muscle tissue stimulation. We specifically selected pressure pain stimulation due to its high test–retest reliability and ecological validity in mimicking deep tissue and musculoskeletal pain (Graven-Nielsen et al. 2017). Pressure stimulation allows for tonic and sustained activation of the deep nociceptors, reflecting chronic pain better than cutaneous paradigms such as cold or heat (Graven-Nielsen and Arendt-Nielsen 2010). Cuff-algometry is safe, user-independent, and provides highly reproducible measures of pain thresholds (Graven-Nielsen and Arendt-Nielsen 2010). These methodological advantages were critical for our study, which focused on pain context modulation under consistent stimulation intensity. The cuff was connected to a compressor and wrapped around the left calf of the participants, at the level of the gastrocnemius muscle. The compression rate of the cuff was controlled by a computer. The maximum pressure limit was 100 kPa (760 mmHg), and a hand-held release button allowed immediate termination of the stimulation. Prior to every session, a calibration of the painful stimulation was done. The pain ratings prior to the auditory test were used to determine the pressure pain during the actual experiment. The pressure pain never exceeded a VAS of 7. The painful stimulus had a fixed pain intensity during the auditory task and depending on the initial condition (NR or PP), the pain stimulus intensity varied based on condition and consequence. In the NR condition, the subject experienced pain relief as a reward and unaltered pain stimulus as a punishment. In the PP condition, the subject experienced increased pain as a punishment and unaltered pain stimulus as a reward. Each pressure stimulation consequence lasted 15 s after the auditory feedback of correct or incorrect answers.

#### Painful stimulation calibration

During the painful stimulation calibration, the cuff was linearly inflated at a 1 kPa/s rate up to 100 kPa. Participants continuously scored the perceived pressure sensation on a computerized visual analogue scale (VAS) ranging from 0 to 10, where 0 represented no sensation, 5 indicated the first pressure at which it became painful (i.e. pressure pain threshold), and 10 represented the most intense pressure pain imaginable (Graven-Nielsen et al. 2017). The pain threshold was based on the average of three measurements during the inflation of the cuff.

### Data analysis

#### Behavioral data

The auditory task performance for each participant was calculated based on the number of correct and incorrect trials. A correct response was defined as all 5 words in the HINT sentences being repeated correctly by the subject, while less than 5 correctly repeated words were considered as an incorrect answer.

#### EEG data pre-processing

EEG data were preprocessed and analyzed using EEGLAB (v.2022.0) (Delorme and Makeig 2004) and Matlab (v.2021b) functions for the extraction of EEG features. For each participant, EEG data were first resampled at 512 Hz. Power-line sinusoidal artifacts between 50 and 100 Hz were removed using Cleanline (https://www.nitrc.org/projects/cleanline). Data were then band-pass filtered between 0.1 and 40 Hz (IIR filter, order 8). Data were then re-referenced to the ear lobes. The independent component analysis (ICA) was then run on the data high pass filtered at 1 Hz to eliminate artefacts due to eye blinks and movements etc. (Makeig et al. 1995). The ICA components were then used to remove unwanted components from the previously filtered data (0.1–40 Hz). Finally, the data were epoched, aligned to the auditory feedback cue, from − 500 to 1000 ms, and baseline corrected (from − 500 to 0 ms). The ERPs were averaged over all trials from the same condition and correct or incorrect answer and analyzed at the Cz electrode (Torta et al. 2019). Three main ERP complexes were semiautomatically (using the findpeaks function on Matlab and visually confirming the correctness of the identification) identified on the Cz electrode and analyzed, namely P1N1 at around 100ms after the stimulus onset, P2N2 at around 200ms and P3N3 at around 300 ms. Scalp topographies of the central electrode (Cz) were created divided by peak, answers, and condition, to check on the activity of the ERPs. To plot the scalp topographies, the noisy channels were removed (In two participants, the F1 channel was removed due to faulty return of signals).

### Statistical analysis

This study is the first to investigate how operant conditioning principles in a reward and punishment context affect ERPs, but an earlier study found a moderate effect size on P300 amplitudes in a reward and punishment paradigm (Zheng et al. 2017). Given the unknowns of how pain and pain relief may influence ERPs, this effect size was conservatively estimated to be low, and with alpha and beta set to 0.05 and 0.80, respectively, a total of 24 participants were needed. To account for possible dropouts, another five participants were included.

A generalized linear mixed model (GLMM) was used to analyze the performance in the HINT task. The correctness of each trial was dichotomized and defined the outcome variable; 5 correctly repeated responses were considered as correct trial, while less than 5 was considered as an incorrect trial. The condition (NR or PP) was considered as categorical factor, and the trial number (1–60) was considered as a continuous factor. A logit link function and a first order autoregressive covariance structure was used. A logit link function was used to model binary response outcomes (correct vs. incorrect), while a first-order autoregressive covariance structure accounted for temporal dependencies between trials, improving model fit in repeated-measures data (Ruíz et al. 2023). A one-way repeated-measures ANOVA was conducted to confirm that the pressure intensity (VAS scores) remained consistent across sessions. Moreover, a two-way RM ANOVA was conducted with two within-subject factors conditions (PP, NR) and answer (correct, incorrect) on the amplitudes of the ERP complexes P1N1, P2N2, and P3N3. In case of an interaction, the estimated marginal means were compared between conditions and between answers. In the case of condition × answer interactions, all multiple comparisons were Bonferroni-corrected. A *p*-value below 0.05 was considered statistically significant. All statistical analyses were performed in Statistical Package for Social Sciences (SPSS; version 24).

## Results

### Participants

Three participants were excluded due to artifacts in the EEG signal and the final analysis was carried out on the 26 remaining participants.

### Behavioral data

There were no performance differences in the HINT trials between the two conditions (*p* = 0.95), but the odds of the total number of correct words repeated increased by 7% (95% CI [1%; 14%], *p* < 0.023) for each 10 trials. The interaction between condition and trial number was not significant (*p* = 0.78).

### VAS levels across time

The VAS scores of the three pressure levels were stable across the sessions. The one-way RM ANOVA for VAS3, VAS5 and VAS7 showed no significant main effect of sessions (VAS3: F_1,2_ = 1.058, *p* = 0.354, partial ƞ^2^ = 0.036; VAS5: F_1,2_ = 1.873, *p* = 0.163, partial ƞ^2^ = 0.063; VAS7: F_1,2_ = 0.775, *p* = 0.466, partial ƞ^2^ = 0.027).

### ERP data

**Fig. 3:**
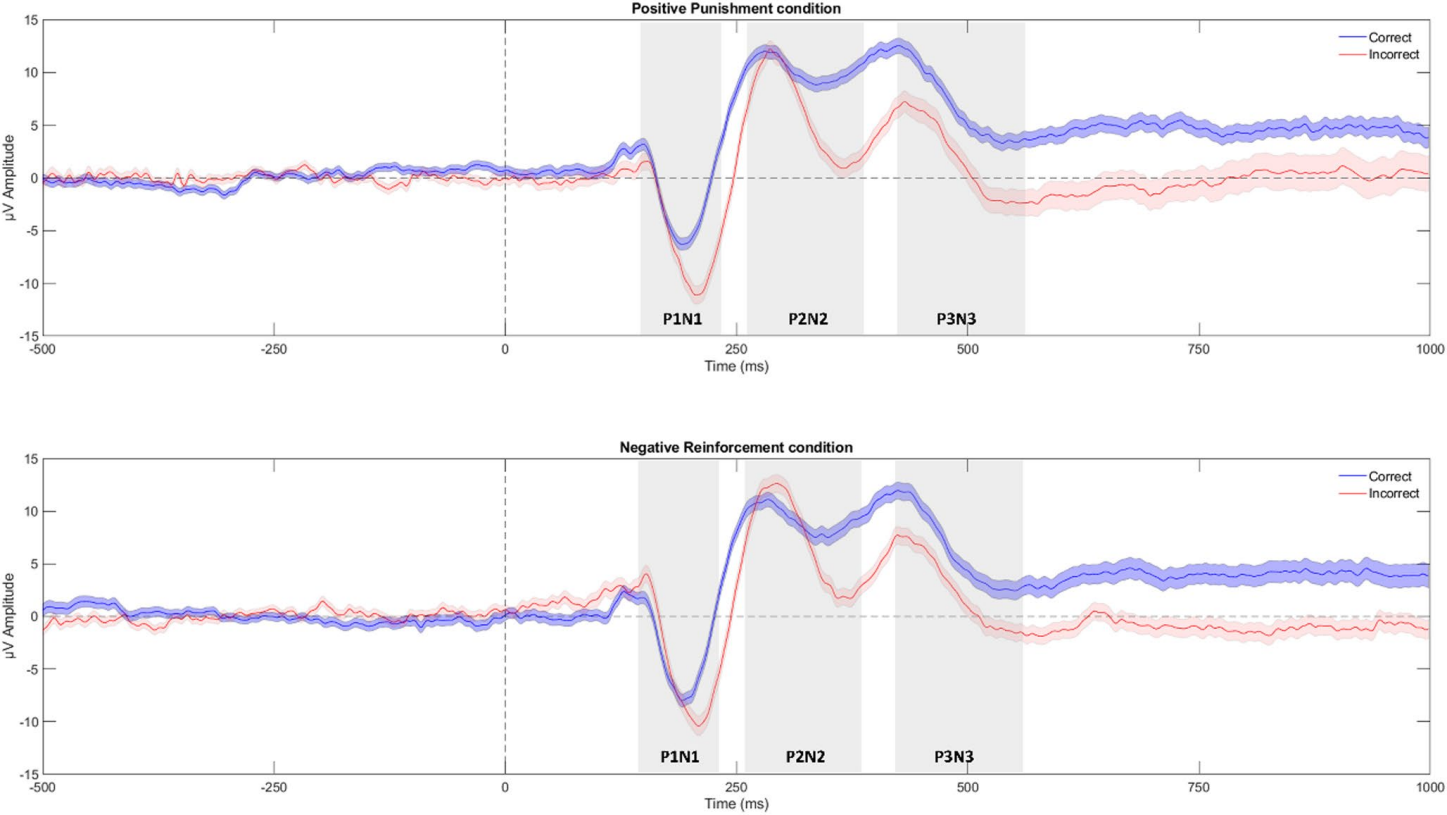
**A** Positive Punishment condition grand mean of all the subjects; **B** Negative Reinforcement condition grand mean of all the subjects. The red lines represent the ERP average of all incorrect answers across the trials. The blue lines represent the average ERP amplitudes of all correct answers across the trials. The envelopes represent the standard error of the mean

**Fig. 4:**
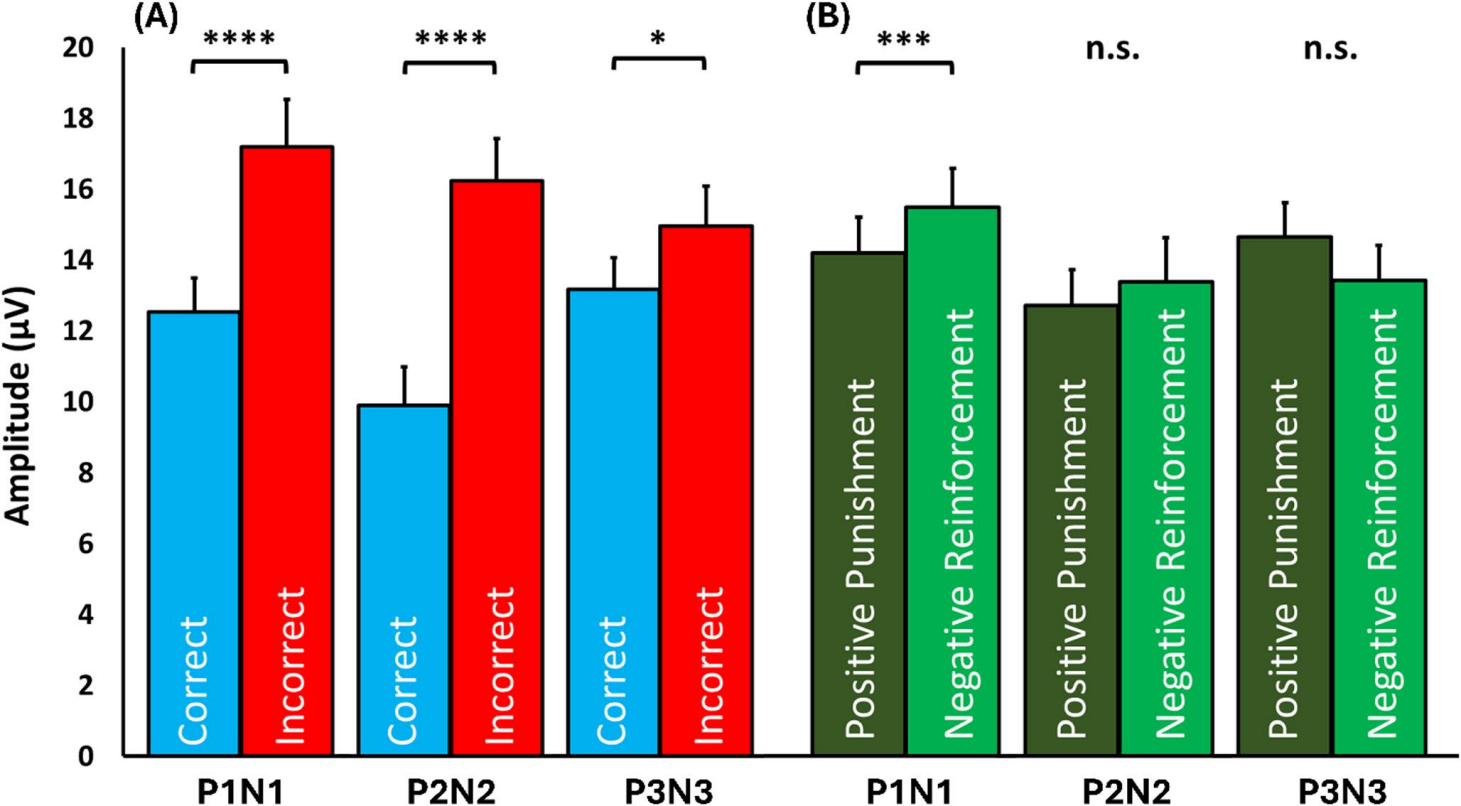
Bar plot representation of the peak-to-peak amplitude average value with the standard deviation of the P1N1, P2N2, and P3N3 complexes for the main effects of the two-way RM ANOVA. **A** When participants received feedback of an incorrect response, a significant increase in peak-to-peak amplitudes were found for P1N1, P2N2, and P3N3. **B** A significant increase in peak-to-peak amplitude was found for P1N1, when participants were in the negative reinforcement condition. Error bars represent standard error of the means

The peak-to-peak amplitude of the ERPs in every complex was higher when the subject responded incorrectly, and therefore expecting a higher painful stimulation afterwards. A graphical representation of the extracted ERPs is shown in Fig. 3. The two-way RM ANOVA for the P1N1 data did not show a significant interaction between condition and answers (F_1,25_ = 0.943, *p* = 0.34, partial ƞ^2^ = 0.036), but main effects of condition (F_1,25_ = 14.338, *p* = 0.001, partial ƞ^2^ = 0.364) and answers (F_1,25_ = 32.294, *p* < 0.005, partial ƞ^2^ = 0.564). The P1N1 mean peak-to-peak amplitude after an incorrect answer (17.19 ± 1.33 µV) was higher than after a correct answer (12.54 ± 0.94 µV) (Fig. 4A). The mean P1N1 peak-to-peak amplitude in the NR condition (15.51 ± 1.16 µV) was higher than in the PP condition (14.23 ± 1.02 µV) (Fig. 4B). For the P2N2 data, there was no significant interaction (F_1,25_ = 0.034, *p* = 0.856, partial ƞ^2^ = 0.001), but a main effect of answers (F_1,25_ = 62.64, *p* < 0.0005, partial ƞ^2^ = 0.715), with higher mean peak-to-peak amplitudes of P2N2 after an incorrect answer (16.34 ± 1.2 µV) compared to after a correct answer (9.89 ± 1.1 µV) (Fig. 4A). No main effect of condition was found for P2N2 (F_1,25_ = 0.83, *p* = 0.371, partial ƞ^2^ = 0.032) (Fig. 4B). No interaction was found for P3N3 ERPs (F_1,25_ = 0.84, *p* = 0.369, partial ƞ^2^ = 0.037), but main effect of answers was significant (F_1,25_ = 5.591, *p* = 0.027, partial ƞ^2^ = 0.203), with higher mean peak-to-peak amplitudes of the P3N3 after an incorrect answer (14.95 ± 1.12 µV) than after a correct answer (13.17 ± 0.89 µV) (Fig. 4A). No main effect of condition was found for P3N3 (F_1,25_ = 2.41, *p* = 0.135, partial ƞ^2^ = 0.099) (Fig. 4B).

### Scalp topographies

**Fig. 5:**
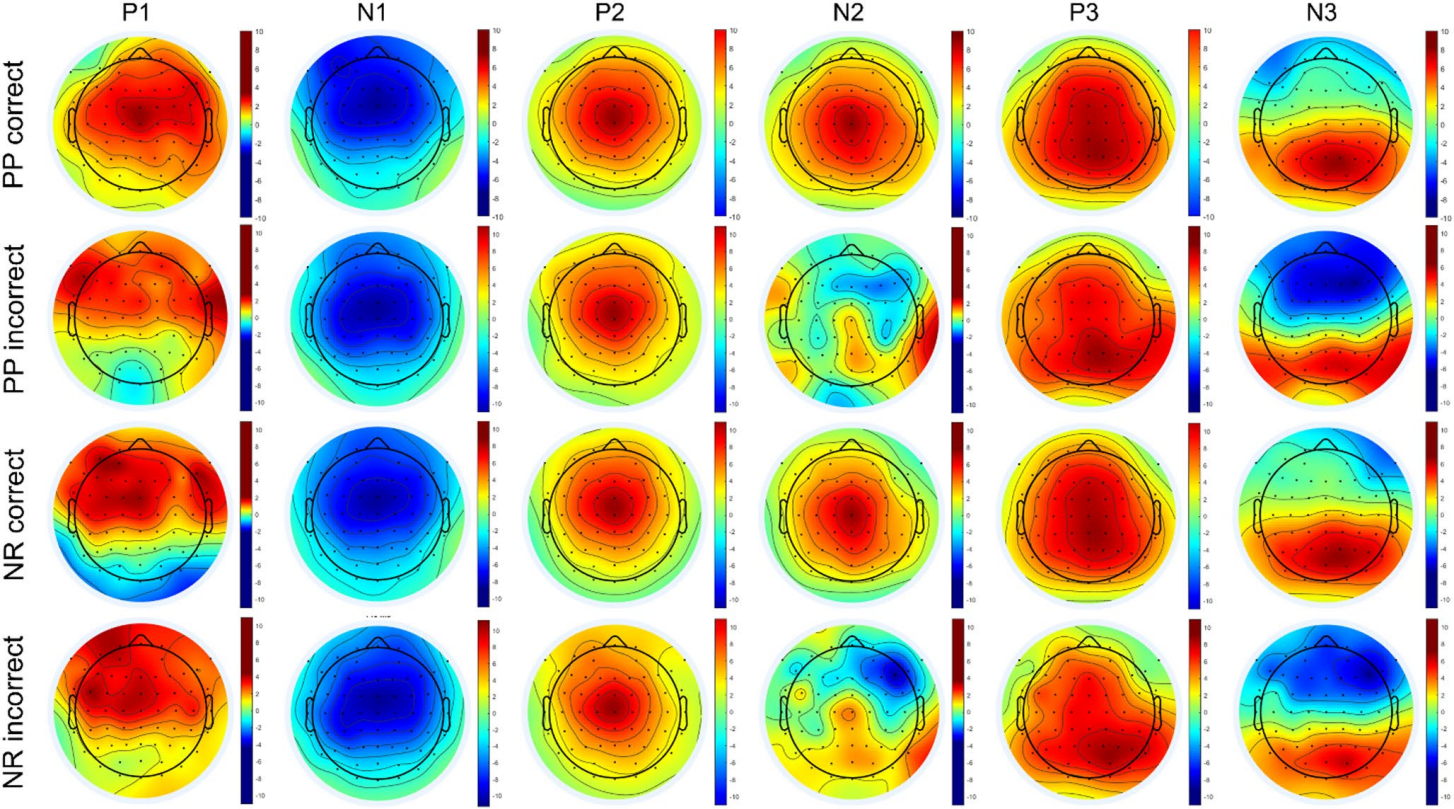
Scalp topographies of the peaks of interest (P1, N1, P2, N2, P3, N3) of the grand average brain activity of the participants across the experiment. The topo plots are divided per initial conditions (PP and NR) and per correct/incorrect answer. NR: Negative Reinforcement; PP: Positive Punishment; P1: P100; P2: P200; P3: P300; N1: N100; N2: N200; N3: N300

The grand-average topographies for each peak analyzed, divided by condition and answers, are represented in Fig. 5. Highest activity for P1, P2, and P3 were found at the central electrode (Cz), showing a positive polarity across both conditions (NR or PP) and answers (correct or incorrect). There was a strong negative polarity for N1 at the central electrode (Cz) in both conditions and answers. Interestingly, for N2, there was a visible difference in polarity between correct and incorrect answers, but not between conditions. For N3, the same tendency towards a stronger negative polarity between correct and incorrect answers can be seen, where the impact of incorrect answer feedback, may be driven by N3 rather than P3.

## Discussion

The present study investigated the effects of pressure stimulation on operantly conditioned auditory feedback ERPs. In contrast with the hypothesis, the constant pressure pain caused an increase in all auditory feedback ERPs when the task performance was incorrect. The P1N1 complex showed a significant increase in the NR compared to the PP condition, whereas no such effect was observed for P2N2 and P3N3. For the behavioral data, both the NR and PP conditions showed non-significant differences in performance scores, but a small learning effect was observed during both conditions.

### P1N1

P1N1 complex amplitude is considered to be regulated by the subjective meaning of the incorrect answer, and in context to the current study, the auditory feedback that pain either will not change or increase is reflected by increased peak-to-peak amplitudes. For instance, the negativity of this complex is generated by part of the posterior medial frontal cortex (pMFC) an area of the brain involved in facing threats (e.g. pain) (Ullsperger et al. 2014) and in helping managing unfavorable situations (Holbrook et al. 2020). In accordance, the current findings suggest that when the feedback indicates an incorrect answer followed by pain as a consequence, the amplitude of P1N1 increases. This may be related to error-driven adaptation to the received feedback (Traxler et al. 2022). A previous study showed that performing a money gain task elicited adaptations, driven by the pMFC, that yielded error corrections (Danielmeier et al. 2011). The prefrontal cortex transmits top-down signals to various brain regions, ultimately shaping behavior (Fuster 2000; Miller and Cohen 2001; Miller and D’Esposito 2005). As an example, the pMFC is involved in action monitoring and is particularly influenced by errors (e.g. incorrect answers), and helps in adjusting performance on a given task immediately or through slower associative learning mechanisms (Ridderinkhof et al. 2004). Considering the present results with these findings, it could be speculated that the pMFC is primarily concerned with predicting the probable outcomes of our actions (e.g. expectation of a painful stimulus) (Alexander and Brown 2011), and may promote performance adjustments. Therefore, future studies should focus on P1N1, especially in relation to incorrect task-related outcome feedback, where pain may function as the consequence of poor performance, which could yield new information on painful punishment and its influence on reward processing. This becomes interesting in relation to chronic pain patients, as they may have heightened processing, as reflected by increased N1 and N2 reactivity to aversive stimuli, when compared to healthy controls (Flor et al. 1997).

### P2N2

P2N2 is a complex that integrates saliency (relevance) and valence (positive or negative) of a given outcome (e.g. the consequence) and is mainly sensitive to incorrect outcomes (Wischnewski and Schutter 2018). P2 alone is usually related to processing of response valence, where an earlier report (San Martin et al. 2010) found higher amplitudes with rewarding answers while others (Carretie et al. 2001, 2005; Schuermann et al. 2012) showed a higher amplitude associated with punishment. In extension, N2 is the negative potential for expected outcomes (Hajcak et al. 2007; Weismüller and Bellebaum 2016). Based on Fig. 3, it is reasonable to hypothesize that the N2 has a larger influence on the responses (correct/incorrect) than P2 during the auditory feedback. Moreover, scalp topographies showed a visible difference in N2 between answers, but not conditions, further strengthening this speculation. When receiving feedback on an incorrect response, the N2 peak decreased, while P2 remained consistent in amplitude, indicating that N2 drives the increase in P2N2. In accordance, an earlier study showed that feedback negativity (N2) increased after an incorrect feedback response and was independent of reward magnitude (Sato et al. 2005). However, conflicting evidence exists, as Wischnewski and Schutter (2018) showed that N2 is insensitive to both reward and punishment. This discrepancy may be explained by the fact that pain was associated with the positive punishment condition, while earlier studies did not elicit pain as the aversive consequence. In this respect, the mesolimbic dopaminergic system is known to influence the generation of P2N2, and has a primary role in reward processing, behavioral analysis, and cognition (Alcaro et al. 2007). The current results therefore suggest that P2N2 has a direct relation to reward and expectation of an aversive consequence. Further research on its relation to specifically punishment feedback is needed to consolidate this finding, and its possible association with the established change in reward processing as found in chronic pain patients (Elvemo et al. 2015).

### P3N3

P3N3 is complex that integrates learning effects and is related to outcome responses and cognitive processing (Wischnewski and Schutter 2018). The present results demonstrate that when subjects answered incorrectly, P3N3 increased in peak-to-peak amplitude as compared to when answering correctly. In contrast, an earlier study suggested that larger reward magnitude increases P3, while not dependent on correct or incorrect answers (Sato et al. 2005). One explanation could be that monetary gain (reward) affects P3N3 differently, as there is no direct physical threat associated with loss of money. As the current study used increased pain or no change in pain as the punishment condition, it is possible that the increase in P3N3 as a response to incorrect answer feedback, is due to the more salient threat of pain and therefore reflects a cognitive realization that more pain will be felt. It is worth noting, that other studies have shown contradicting results, where reward was associated with an increase in P3 (Peterburs et al. 2013; Wischnewski and Schutter 2018). It could be speculated that the reason for the discrepancy in findings is that pain relief decreases the need for cognitive integration of outcome responses, which may not be the case for monetary gain. Future research could investigate the P3N3 response to monetary gain versus pain relief, to determine if cognitive outcome processing is different depending on the type of reward.

### Behavioral performance data of the HINT

The performance scores were not affected by the type of condition (NR or PP). However, the data suggested the presence of a small odds improvement in performance score across trials. This was unexpected as the study design aimed at a 50–50% distribution of correct and incorrect answers. However, an earlier study reported that reward may increase error adaptation, which in turn may explain our findings and that feedback of an incorrect answer resulted in stronger adaption towards completing the consecutive HINT tasks correctly (Stürmer et al. 2011). Another explanation may be habituation to the white noise, since an earlier study demonstrated that individuals may habituate to acoustic white noise during cognitive and perceptual tasks, but is not generalized, and highly task specific (Herweg and Bunzeck 2015). Other studies may investigate how changing the distribution of correct and incorrect answers may influence auditory-feedback related ERPs in the context of operant conditioning.

### Clinical implications of the operant conditioning model in chronic pain management

Operant conditioning has been widely used in physical therapies for chronic pain rehabilitation (Gatzounis et al. 2012). For instance, Graded Activity has proven effective for conditions like low back pain and chronic fatigue syndrome, outperforming standard care and no intervention (Lindström et al. 1992; White et al. 2011). Moreover, a previous study have shown, that eight weeks of cognitive behavioral therapy (based on operant conditioning principles) can increase, blunted late positive potential reward responses which in turn can predict chronic pain severity (Garland and Howard 2018). The present study adds to the existing literature by showing that in the context of painful pressure stimulation, operant conditioning of the feedback on performance of an auditory task, leads to increased P1N1, P2N2, and P3N3 amplitudes, and improves odds of a successful auditory task performance. An interesting prospect for future research may be to prolong the experimental protocol, and include e.g. Graded Activity as an intervention, to investigate how it affects auditory feedback-related ERPs.

### Limitations

The current findings are based on a novel approach, using pain as reinforcement and punishment in operant conditioning. Nonetheless, certain limitations should be considered when appraising the results. The hypothesis of reduced ERPs was based on prior work showing that e.g. acute experimental pain can attenuate cortical ERPs such as N2/P2 (Valeriani et al. 2005; Willems et al. 2021) and was extended to the current study. This may have been unjustified, as the broader literature on ERPs and aversion often report increased amplitudes of single peak values N1 and P3 (Cui et al. 2016), and N1 and P2/P3 (Willems et al. 2021), with some conflicting results (Ma et al. 2022). Moreover, cortical reward and punishment responses have traditionally been investigated using peak ERP values which reflect distinct neural processes associated with the cognitive, sensory, and emotional processing of information (Meyer et al. 2021). The current study used the peak-to-peak amplitudes as they reflect the integration of various parameters. Peak amplitudes alone are known to come with several disadvantages when considering response patterns, where peak amplitude, e.g., is not robust in a setting with background noise which is known to affect EEG recordings to a substantial degree (Clayson et al. 2013; Luck 2005). Moreover, peak amplitude is measured by subtracting the baseline or the zero point of the signal from the actual voltage value of the peak and is therefore affected by baseline stability. The use of peak-to-peak amplitudes in the current study also addresses earlier studies that show differences in how individuals perceive e.g. punishment and reward, where those who are highly sensitive to punishment, show stronger motivation and engages more to avoid punishment (Boksem et al. 2008), which is nullified as peak-to-peak is relative to a participant’s own sensitivity. While both peak analysis and peak-to-peak present with issues (Clayson et al. 2013), the peak-to-peak analysis ensures that we can infer on integration of cognitive and sensory information.

## Conclusions

The current results have demonstrated that the amplitudes of P1N1, P2N2, and P3N3 complexes increased after an incorrect answer. Future research may investigate how such error-driven increases in auditory-feedback related processing are associated with the type of stimulus used, delivery method, and its transferability to chronic pain conditions.

## Data Availability

No datasets were generated or analysed during the current study.
